# Evolutionary Pressure on Mitochondrial Cytochrome *b* Is Consistent with a Role of CytbI7T Affecting Longevity during Caloric Restriction

**DOI:** 10.1371/journal.pone.0005836

**Published:** 2009-06-08

**Authors:** Wesley A. Beckstead, Mark T. W. Ebbert, Mark J. Rowe, David A. McClellan

**Affiliations:** 1 Department of Biology, Brigham Young University, Provo, Utah, United States of America; 2 Department of Nutrition, Dietetics, and Food Science, Brigham Young University, Provo, Utah, United States of America; Roswell Park Cancer Institute, United States of America

## Abstract

**Background:**

Metabolism of energy nutrients by the mitochondrial electron transport chain (ETC) is implicated in the aging process. Polymorphisms in core ETC proteins may have an effect on longevity. Here we investigate the cytochrome *b* (cytb) polymorphism at amino acid 7 (cytbI7T) that distinguishes human mitochondrial haplogroup H from haplogroup U.

**Principal Findings:**

We compared longevity of individuals in these two haplogroups during historical extremes of caloric intake. Haplogroup H exhibits significantly increased longevity during historical caloric restriction compared to haplogroup U (*p* = 0.02) while during caloric abundance they are not different. The historical effects of natural selection on the cytb protein were estimated with the software TreeSAAP using a phylogenetic reconstruction for 107 mammal taxa from all major mammalian lineages using 13 complete protein-coding mitochondrial gene sequences. With this framework, we compared the biochemical shifts produced by cytbI7T with historical evolutionary pressure on and near this polymorphic site throughout mammalian evolution to characterize the role cytbI7T had on the ETC during times of restricted caloric intake.

**Significance:**

Our results suggest the relationship between caloric restriction and increased longevity in human mitochondrial haplogroup H is determined by cytbI7T which likely enhances the ability of water to replenish the Q_i_ binding site and decreases the time ubisemiquinone is at the Q_o_ site, resulting in a decrease in the average production rate of radical oxygen species (ROS).

## Introduction

Mitochondria have long been implicated in the aging process [1]–[4]. The electron transport chain (ETC), embedded within the inner membrane of the mitochondria, is the major producer of reactive oxygen species (ROS), which are presumed to be the primary agent for cell damage and premature apoptosis, affecting aging and longevity [5]–[8]. The primary intermediate responsible for producing the ROS superoxide is ubisemiquinone, the coenzyme Q (CoQ) radical produced in complexes I, II and III of the ETC [9]. Reducing the production of ubisemiquinone in the ETC has been shown to reduce free radical levels and prolong life span in animals [10], [11].

The mechanism by which ROS affect aging and longevity has recently come under scrutiny (e.g. [12]–[14]). For years the paradigm of aging (e.g. [15]–[20]) has predicted that over time ROS leakage leads to accumulation of mitochondrial DNA (mtDNA) mutations and oxidative damage to the cell. Over the lifespan of an individual, the damage may then lead to premature cell death, followed by organ and tissue failure, which are characteristics of aging and associated degenerative disorders. The paradigm concludes that to avoid cumulative damage over time and increase longevity, antioxidants must be taken to combat oxidative stress on cell components.

Recent studies indicate that these basic assumptions should be revisited. It has been shown that free radical leakage fluctuates according to the signals that ROS themselves produce [12], [13]. Further, antioxidants have been shown not to prolong lifespan (e.g. [14], [15]). Together, the ROS fluctuations and the lack of an antioxidant effect on longevity indicate that the role of free radicals in aging and longevity is more complex than previously thought.

A theory of aging that accounts for a signaling role for endogenous free radicals in maintaining the metabolic status of the cell has been proposed [12], balancing their role in cellular damage. The underlying principles of this theory of aging include: 1) ROS leakage produces mtDNA mutations; 2) ROS produced by ailing mitochondria also signals cellular apoptosis activities; 3) when a threshold of both ailing mitochondria and ROS signals is reached, the cell prematurely commits to apoptosis followed by organ and tissue failure.

Studies have shown that when a given mutation is found in different species it has varying effects based on the comparative rate of ROS leakage in the species [16]. The threshold of ROS signal needed for the cell to commit apoptosis depends on the rate at which ROS are produced from the mitochondria. Intuition might suggest that the threshold of ROS signal needed for the cell to commit to apoptosis is static. However, it appears to be dependent upon the rate of ROS production from the mitochondria, exhibiting a tight correlation between mutations and a ROS-signal apoptotic threshold [17].

Whether the role of free radicals in aging and longevity involves the toxicity of ROS over time or the important signaling role of ROS in programmed cell death, it is important that studies of longevity turn attention toward mechanisms by which ROS is produced in the respiratory chain of the mitochondria, and how leakage affects the cell and might be reduced.

In this regard, it is also essential to come to an understanding of these mechanisms in the context of caloric intake, since electron input to the ETC may alter ROS production [8]. It has been proposed that as mitochondria function in decreased phosphorylating modes, the ETC remains in a more reduced state (maximally occupied with electrons) for longer time periods, increasing the production of ubisemiquinone and ROS, thereby decreasing longevity [8], [10], [18]. The major contributor toward a more reduced state is excessive calorie consumption (increased electron input), but other factors can exacerbate the problem as well. For example, the ETC may remain more reduced because of inhibition or dysfunction of ATP production via oxidative phosphorylation, blocking the electron flow of the respiratory chain. Reduced ADP, caused by a lack of physical exercise (during which ADP is not present because ATP is not being used), may also inhibit the turning over of electrons and keep the ETC more reduced. As the electron flow is inhibited, not only do more reactive electrons accumulate, but oxygen levels increase as well. This may increase the probability that backed up electrons and oxygen will react and produce free radicals.

The electron transport chain is composed of protein complexes whose individual protein subunits are encoded in either nuclear or mitochondrial DNA. Nonsynonymous single nucleotide polymorphisms (SNPs) in any of the genes encoding ETC subunits could alter the quality of electron flow or affect CoQ binding sites, and subsequently affect ROS production, aging and longevity.

Mitochondria have maintained a core set of genes that encode essential proteins in the ETC. Nonsynonymous mutations in these genes have the potential to affect the ETC, ROS production and longevity in a way that is dependent upon calorie restriction and/or calorie over-consumption. Throughout the last 150 years there have been dramatic extremes in per capita caloric intake. For example, during the Great Depression (1920–1940) many individuals throughout North America were under extremely restricted caloric intake. In more recent decades there has been an increase in caloric intake toward the other extreme, particularly in North America. If there is a relationship between the redox state of the ETC, longevity, mtDNA mutations and extremes of caloric intake, it could be demonstrated by an analysis of historical longevity within mtDNA haplogroups during extended and continental periods of calorie reduction and over-consumption.

The human population is subdivided into mitochondrial haplogroups. Haplogroups are distinguished by a unique set of mitochondrial SNPs, the nonsynonymous of which are of interest in relationship to their potential effect on the mitochondrial respiratory chain and longevity. Many studies have demonstrated the association of certain mtDNA haplogroups with increased longevity (e.g. [19]–[21]). We chose to focus on haplogroup H, which is one of the more recent haplogroups, but also now the most prevalent European mtDNA haplogroup, and compare historical longevity in closely related haplogroup U individuals under extremes of caloric intake.

**Figure 1:**
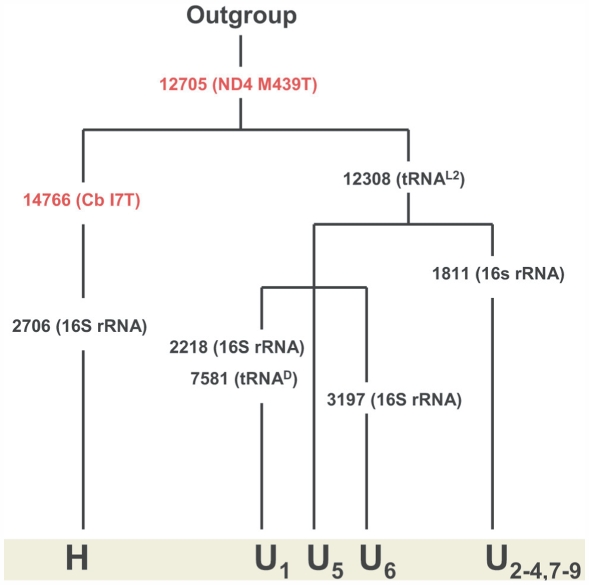
Haplogroup relationship for H and U. The mitochondrial SNPs that separate them are shown. Non-synonymous SNPs are shown in red. RNA gene mutations are shown in black. The data was obtained from MITOMAP (http://www.mitomap.org) [41], [42]. The only protein-coding SNP that separates the two haplogroups and that is therefore analyzed in this study is T14766C, which results in an amino acid substitution of a threonine for an isoleucine at site 7 in cytochrome *b*.

Figure 1 shows the haplogroup relationship with regard to mtDNA mutations between H and U. Haplogroup H is separated from haplogroup U by mitochondrial SNP T14766C, which results in an amino acid substitution of a threonine for an isoleucine at amino acid site 7 in cytochrome *b* (cytb), which encodes the central catalytic enzyme of the mitochondrial protein complex III (cytochrome *bc_1_* complex) of the ETC.

In this study we first identify the difference in longevity in haplogroups H and U specifically during times of caloric restriction as well as times of calorie over-consumption. This is accomplished by identifying individuals with haplotype H and U from two genetic genealogy databases and collecting longevity information about their maternally related ancestors from the pedigrees in those databases. In addition, many of the ancestors were then found in a family history database, and longevity information was gathered about their extended maternal relatives who share their maternally inherited haplogroups H and U. The longevity data for haplogroups H and U were compared in cohorts of 20 year increments, with 1920–40 longevity representing historical calorie restriction, and 1960–80 and 1980-present representing caloric over-consumption.

Next, we examined the biochemical shift that the polymorphism cytbI7T produces in cytb, protein complex III, and the ETC overall. We then estimate selective pressure on amino acid properties throughout mammalian evolution, particularly at site 7 in cytb, to gain a historical evolutionarily context of this region and a better perspective on how this recent human polymorphism may change the mitochondrial system. Finally, we correlate these data and present a mechanism by which cytbI7T may affect ETC efficiency and ROS production by complex III, and consequently longevity in haplogroup H during a restricted dietary environment compared to an environment of excessive caloric intake.

## Materials and Methods

### Haplotype and Longevity Data Collection

The haplotypes and life spans of individuals who lived between 1870 and the present were collected from Family Tree DNA (http://www.familytreeDNA.com) and the Sorenson Molecular Genealogy Foundation (http://www.smgf.org). The female individuals whose haplogroup was identified from these databases were used to identify maternally related pedigrees from Family Search (http://www.familysearch.org). Data for 737 individuals from Haplogroup H and 890 individuals from Haplogroup U were collected and evaluated.

Individuals were included if they met the following criteria. First, only females were included to avoid the differences between female and male longevity. Second, individuals were included if they died in North America. This criterion was chosen in order to increase the probability that individuals included in this study lived under somewhat similar caloric environments. Finally, the individuals were included only if they died at or after the age of 60. This was done to increase the probability that included individuals died from age related factors including degenerative disorders rather than by accidents or other unrelated causes.

The human population has undergone dramatic shifts in caloric intake during different time periods throughout the last 200 years. In order to elucidate the impact of caloric intake on longevity, individuals were grouped together by the time period in which they died. Table 1 shows the number of individuals from each of the two haplogroups H and U in each time period.

**Table 1:** The number of individuals used in this study from each haplogroup during each time period.

Time Period	Haplogroup H	Haplogroup U
1870–1920	302	286
1920–1940	88	121
1940–1960	101	151
1960–1980	154	207
After 1980	92	125
**Total**	**737**	**890**

### Biochemical and Evolutionary Correlation

As shown in Figure 1, haplogroup H is separated from haplogroup U and other haplogroups by mitochondrial SNP T14766C, which results in an amino acid substitution of a threonine for an isoleucine at site 7 in cytb, thus possibly affecting protein complex III and the ETC. In order to computationally generate information about the possible physicochemical effects of this SNP on cytb, respiratory chain efficiency, and longevity in Haplogroup H individuals, the analytical program TreeSAAP [22]–[24] was used to examine the following:

- The effect(s) cytbI7T may have on the physicochemical properties of the N-terminal region of the cytb protein.
- The naturally occurring effects of selection on the physicochemical properties of the N-terminal region of the cytb protein over the phylogenetic history of mammals – to establish a context within which to interpret the broader scope of the human SNP cytbI7T.

The details of these two steps will be discussed in the remainder of this section.

TreeSAAP was originally developed to detect molecular adaptation due to natural selection across a protein sequence by statistical analysis of the amino acid substitutions across a phylogenetic tree. This adaptation is expressed in terms of amino acid property changes. TreeSAAP measures the physicochemical magnitude of amino acid substitutions and indicates which amino acid properties have likely been affected by natural selection during the evolutionary process.

As the first step, we modified the use of TreeSAAP, allowing the program to analyze the physiochemical changes produced by a single amino acid substitution [25]. TreeSAAP was thus used to detect the radical physicochemical shifts that are produced by a mutation substituting a threonine for an isoleucine at site 7 in cytb of mitochondrial complex III. TreeSAAP was implemented by grouping changes into one of eight magnitude categories, 1 being the most conservative and 8 being the most radical. In this study we chose to focus on amino acid property changes of category 6, 7, and 8 because they unambiguously indicate a significant change in the protein [24].

In the second step, the entire protein-coding portion of the mitochondrial genome (13 complete coding sequences) was collected for 107 mammalian species, including sequences from all major mammalian lineages, from GenBank (Supplementary Table S1). These sequences were aligned and used for phylogenetic analysis (Supplementary Figure S1). The cytb gene sequences for the 107 mammalian species and the phylogenetic tree created from all 13 mtDNA genes were used as input for TreeSAAP as in Chamala et al. [26].

We used TreeSAAP to gain a historical context of the amino acid property changes at site 7 in cytb. Specifically, the property changes that TreeSAAP detected in step 1 of the analysis were correlated with the same properties TreeSAAP detected to be under natural selection throughout mammalian evolution. The overall purpose of this second step is to establish context for properly interpreting the results of step one. By estimating the naturally occurring pattern of adaptation, the effects of SNPs may be compared to the location and effects of extant genetic variation and historical adaptations within the broader taxonomic group. When a SNP fails to share characteristics with the historical adaptations of the group, that SNP is more likely have a detrimental effect. Conversely, if a SNP has much in common with the adaptations of the group, that SNP may have a similar adaptive effect [26].

## Results

### Difference in Longevity between H and U

**Figure 2:**
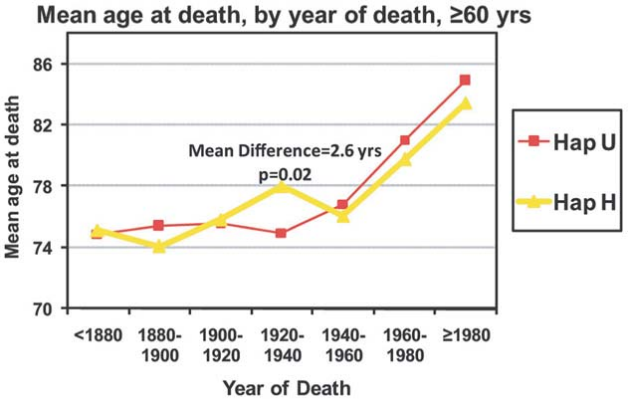
The mean age at death of individuals grouped by time period. Tests for statistical difference were performed using a two-tailed t-test. Haplogroup U is labeled in red and Haplogroup H is labeled in yellow. Haplogroup H shows significantly increased longevity during 1920–1940.

The mean age at death was calculated for each time period cohort of individuals. To test for statistical difference between Haplogroup H and U, a two-tailed t-test assuming equal variances was performed on the means. Figure 2 shows the mean age at death for each time period cohort. We see an expected general increase in longevity during the 20^th^ century in both haplogroups. Before 1920 there is no significant difference between the longevity of individuals in haplogroup H and U. During the caloric restriction of the Great Depression, 1920–1940, haplogroup H shows significant increase in longevity compared to haplogroup U (mean difference = 2.6 years, *p* = 0.02).

**Figure 3:**
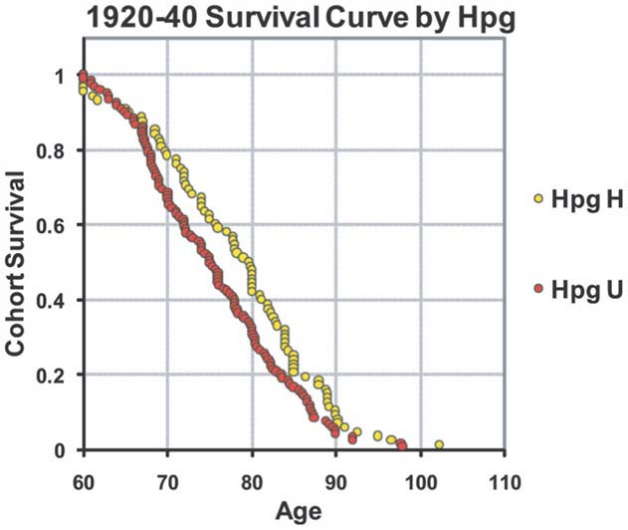
The survival curve for individuals who died after the age of 60 between 1920 and 1940. Haplogroup U individuals are labeled in red and Haplogroup H individuals are labeled in yellow. The graph shows the fraction of the time period cohort that survived (y axis) to a given age (x axis).

This significant difference during caloric restriction is further illustrated by a survival curve of individuals in the two haplogroups during this two decade time period (Figure 3). Following 1940, there is little difference in longevity between Haplogroup H and U individuals. This lack of difference continues to include recent years of caloric over-consumption.

### CytbI7T TreeSAAP Results

Table 2 shows the TreeSAAP results for the single substitution cytbI7T. TreeSAAP indicated that substituting a threonine for an isoleucine at site 7 in cytb results in radical shifts in seven amino acid properties. The large number of properties of magnitude 6–8 (radical to extremely radical changes) that are associated with this polymorphism suggests a radical change in the resulting protein. These properties will be described in detail.

**Table 2:** TreeSAAP results for cytbI7T.

Amino Acid Property	Magnitude Category	Direction of Change
Surrounding Hydrophobicity	8	Decrease
Hydropathy	6	Decrease
Equilibrium Constant (Ionization of COOH)	6	Increase
Average Number of Surrounding Residues	6	Decrease
Buriedness	6	Decrease
Long-Range Non-Bonded Energy	6	Decrease
Solvent Accessible Reduction Ratio	6	Decrease

The amino acid property changes, the radicality of the change, and the direction of the change are shown.

Three properties relate to the level of hydrophilicity in the region. In general, the amino acid property “Surrounding Hydrophobicity” refers to the tendency for the region around the amino acid site in question to interact with water. In the case of cytbI7T, there is a radical decrease of magnitude 8 in this property, indicating that the region surrounding site 7 becomes less hydrophobic and more hydrophilic by introducing a threonine instead of an isoleucine. This biochemical shift is likely the result of the hydroxyl group on the R-group of threonine that may easily interact with water molecules. Isoleucine is hydrophobic and thus lacks this ability. Hydrophobicity is similar to the property “Hydropathy”, which also decreases due to the polymorphism I7T.

“Equilibrium Constant” deals with the ability of any ionizable functional group of the residue to dissociate and make an ion [27]–[29]. TreeSAAP indicates that cytbI7T increases this property by a moderately radical (magnitude 6) change. An increased equilibrium constant for the ionization of COOH would indicate a more product-driven reaction. This would, as in the other properties, make the region more water-soluble and hydrophilic. As will be noted, this may be of greatest importance regarding reduced ROS production and increased longevity during calorie restriction.

The other four properties relate to the level of compactness in the region. The amino acid properties “Average Number of Surrounding Residues” and “Buriedness” measure how compact and buried the amino acid site is. TreeSAAP indicated that cytbI7T decreases both of these properties with a magnitude 6 change. With a decrease in these properties, we can imagine a region around site 7 that is more open and free to interact (as opposed to compact).

This conclusion is further supported by the decrease in the amino acid property “Long-range non-bonded energy” resulting from cytbI7T. This property describes the interactions between molecules that are not directly in contact with one another (such as Van der Waals interactions), affecting the stability of molecules involved. It has been shown that the structures in globular proteins are influenced not only by local, bonding interactions, but also by long-range interactions [29], [30]. TreeSAAP indicated a radical decrease in this property of magnitude 6. A decrease in “Long-range non-bonded energy” indicates a decrease in stability in this residue and surrounding residues, further suggesting that the region around site 7 is more open, less globular, and less compact due to this polymorphism.

The solvent accessible surface of a protein is the region where solvent and solutes interact with the protein [31]. The amino acid property “Solvent Accessibility Reduction Ratio” is defined as the ratio of the solvent accessible surface area of a residue in the native state to that of the residue in an extended tri-peptide (Ala-X-Ala) conformation [27]–[29].

All of these property changes involve either an increase in hydrophilicity or a decrease in compactness in the region surrounding site 7 in cytb among haplogroup H individuals.

### Mammalian Evolution TreeSAAP Results

The second part of our analysis involved the use of TreeSAAP across the cytb sequences and phylogenetic tree of 107 mammalian species from all major mammalian lineages in order to estimate a historical evolutionary context of the amino acid properties that have been under selection for radical change at site 7 in cytb. Particularly, the amino acid properties that are changed by the cytbI7T polymorphism itself (Table 2) were inspected in the TreeSAAP results of 107 mammalian cytb sequences.

**Figure 4:**
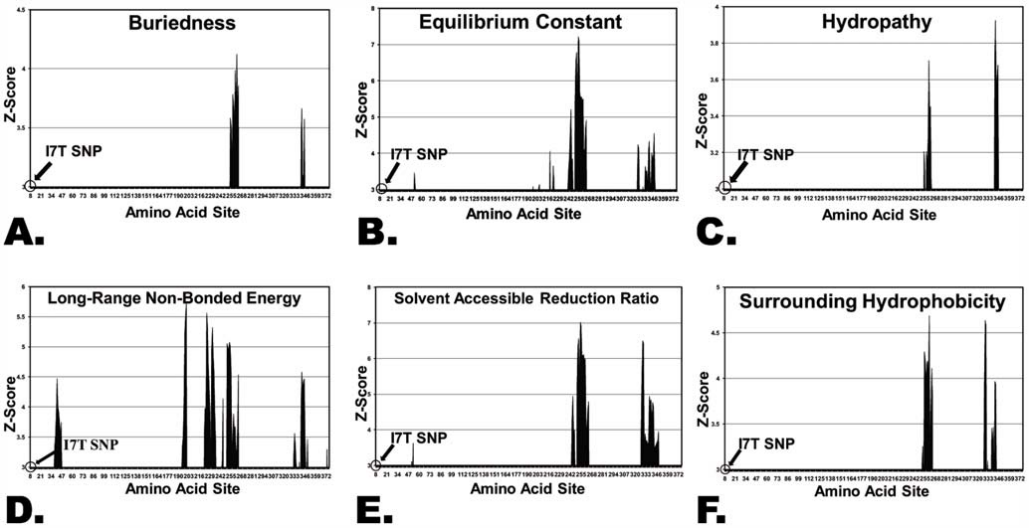
Mammalian evolution TreeSAAP results for the cytochrome *b* protein subunit. Each graph shows evolutionary selection for a given amino acid property across the amino acid sequence of cytb. A z-score above 3.09 indicates radical changes occurring for the given property throughout the evolution of 107 mammalian species (see Materials and Methods). The amino acid site number 7 where the cytb polymorphism occurs is annotated. (A) Shows the TreeSAAP results for the property “Buriedness”. (B) Shows the results for property “Equilibrium Constant”. (C) and (D) Show results for “Hydropathy” and “long-Range Non-Bonded Energy” respectively. (E) and (F) Depict the graphs for the TreeSAAP results of the properties “Solvent Accessible Reduction Ratio” and “Surrounding Hydrophobicity”.

Figure 4 shows the TreeSAAP results across cytb that are consistent with the amino acid properties found by running the individual cytbI7T polymorphism through TreeSAAP. Though TreeSAAP found radical shifts in 7 amino acid properties while analyzing the individual cytbI7T polymorphism, only six of these were significantly affected in the mammalian data set – TreeSAAP did not detect evidence of adaptation for the property “Average Number of Surrounding Residues”.

The peaks in the graphs of Figure 4 represent radical changes in those properties during phylogenesis at particular amino acid sites. Site 7 in cytb, where the polymorphism that distinguishes haplogroup H occurs, are marked in each graph. There are no peaks in any of these graphs at site 7, though there have been naturally occurring radical changes in these properties elsewhere along the sequence. This indicates that the 7 properties affected by cytbI7T have been highly conserved and stable at this site throughout the evolution of these 107 mammalian species.

TreeSAAP results suggest that the region around site 7 likely was not a site of adaptation in hydrophilicity and compactness throughout mammalian evolution. This result, however, does not preclude positive selection at this site in the human lineage. Threonine was either fixed or maintained at cytb site 7 until a T7I mutation occurred that led to an isoleucine polymorphism in primates. Subsequent primates, including most human mitochondrial haplogroups, still exhibit isoleucine at site 7. Relatively recently, the human substitution cytbI7T occurred, forming haplogroup H. Radical physicochemical shifts in haplogroup H restore the historical pre-primate character state, suggesting that cytbI7T may have a positive effect. Given its location within protein complex III and the radical nature of the physicochemical effect, we are led to conclude that cytbI7T was likely advantageous relative to the precise efficiency of complex III and the respiratory chain that is linked to increased longevity during calorie restriction.

## Discussion

The I7T polymorphism analyzed in this study is located in the cytb protein subunit. Cytb is encoded by mitochondrial DNA and is located centrally in complex III (cytochrome *bc_1_* complex) of the ETC. Complex III is a dimer enzyme embedded in the inner membrane of the mitochondria. The complex couples electron transfer from ubiquinol to cytochrome *c* (cytc) with proton translocation across the membrane to contribute to an increased proton concentration in the intermembrane space of the mitochondria [32]–[34]. The resulting proton gradient drives ATP synthesis via oxidative phosphorylation by ATPase (protein complex V).

**Figure 5:**
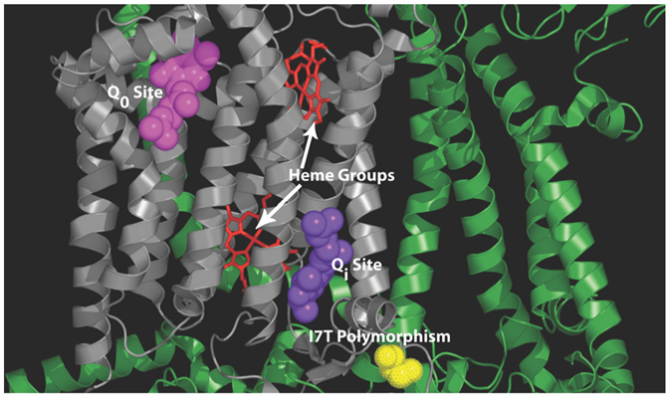
Cytochrome *b* within the cytochrome *bc_1_* complex. Only one monomer is shown. The cytochrome *b* subunit is shown in grey with surrounding subunits shown in green. The CoQ coenzymes are shown bound in the outside (Q_o_) and inside (Q_i_) binding sites. The heme groups that are involved in the transfer of the electrons between binding sites are also shown. The cytbI7T polymorphism (yellow) is located on the N-terminus tail of cytb near the Q_i_ site. The three-dimensional coordinates of the complex were obtained from the protein data bank (http://www.pdb.org) [43] under the entry 1ntz [35]. The structure was visually rendered with PyMOL [44].

The coupling of ubiquinol and cytc is accomplished by the Q cycle, which involves oxidation of ubiquinol at the Q_o_ binding site to create ubiquinone, which is then reduced at the Q_i_ binding site [35]–[37]. Both of these binding sites are located in the protein subunit cytb situated within the hydrophobic center of the inner membrane (Fig. 5). The cytbI7T polymorphism is located on the N-terminus tail of cytb, near the Q_i_ binding site.

**Figure 6:**
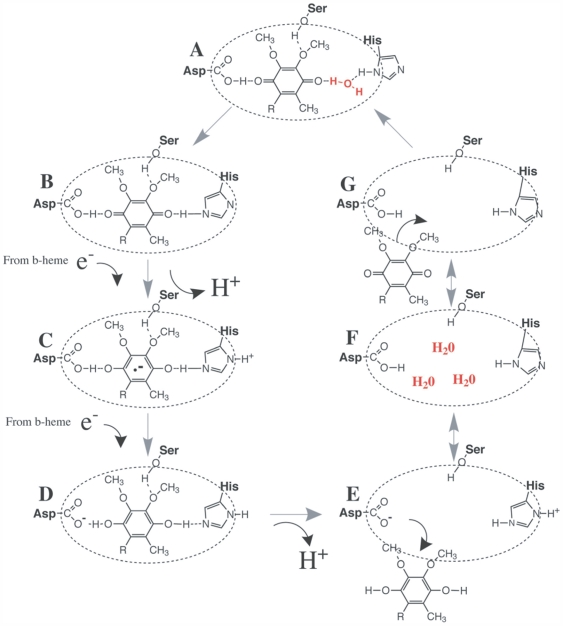
The reduction of ubiquinone to ubiquinol in the Q_i_ binding site in cytochrome *b*. (A) Shows ubiquinone bound in the Q_i_ pocket to surrounding cytb residues. A water molecule involved in H-bonding is highlighted in red. The reduction of ubiquinone proceeds from (B) to (D) with the vacation of ubiquinol shown in (E). (F) Shows the vacant Q_i_ site with replenished water molecules (red) that replace the H^+^ used in the reaction. The cycle continues in (G) with another ubiquinone entering the binding site and the process starts again. The figure was adapted from Kolling et al. [38] and Crofts [36].

Ubiquinone is reduced to ubiquinol at the Q_i_ binding site [36], [38] (Fig. 6). Two important aspects of this reaction are: 1) the presence of water and ubiquinone within the binding site, and 2) water molecules filling the binding site after ubiquinol vacates. High-resolution structures of protein complex III show that water is involved in hydrogen bonds between the quinone and surrounding cytb residues [39], [40]. Water is also replenished in the vacant binding site to replace the H^+^ used in the reduction reaction [36]. Therefore, water molecules are an essential ingredient for the ubiquinone to ubiquinol cycle at the Q_i_ site, and an initial step in proton translocation across the inner mitochondrial membrane.

**Figure 7:**
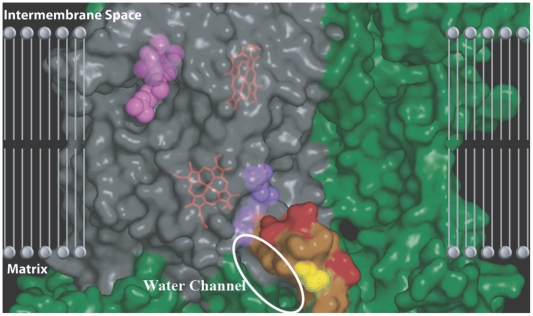
The water channel that leads up into the hydrophobic membrane region where the Q_i_ binding site is embedded. The approximate location of the membrane was obtained from Gao et al. [35]. The electrostatic surface is shown for the residues of complex III (only one monomer shown). Cytb is colored in grey and the surrounding subunits are shown in green. The CoQ coenzymes (pink and purple) are shown bound in their respective sites. The N-terminus tail of cytb has been highlighted according to the hydropathy of the residues in order to show the potential path for water to be shuttled up into the Q_i_ site. Hydrophilic residues on the cytb tail are highlighted in orange and hydrophobic residues are highlighted in red. The figure shows a potential hydrophilic channel up the cytb tail that allows water into hydrophobic membrane and into the Q_i_ site. The cytbI7T polymorphism (yellow) resides in the center of this water channel.

As mentioned, the CoQ binding sites are embedded within the globular complex and the hydrophobic inner membrane. Given this information, we suggest it is not trivial to replenish the essential H_2_0 molecules in the Q_i_ binding site. A careful look at the region surrounding the binding site sheds light on this problem. The three-dimensional structure reveals a hydrophilic region that could potentially be a channel for water to be shuttled into the Q_i_ binding site that is embedded within the globular complex in the membrane (Fig. 7).

The cytbI7T polymorphism lies at the heart of this water channel and likely has an impact on the ability of water to replenish the Q_i_ site because threonine may bind a water molecule with its hydroxyl group, whereas isoleucine lacks this ability. TreeSAAP indicates that the cytbI7T mutation radically changes seven amino acid properties that have to do with either increasing the hydrophilicity or reducing the compactness in the area. Increasing hydrophilicity and making the area more open and less globular in the cytb N-terminal region, water will likely be more attracted to the shuttle area and reach the Q_i_ binding site more easily. This would increase the efficiency of the binding site and the Q-cycle overall, thereby decreasing the time that ubisemiquinone exists at the Q_o_ site, on average decreasing ROS production. To corroborate this idea, TreeSAAP showed that these seven amino acid properties have been highly stable and conserved throughout mammalian evolution in the region surrounding the cytb N-terminal region prior to the T7I SNP in the basal primate branch. With the reintroduction of cytbI7T that created the mitochondrial haplogroup H branch of humans, which is most prevalent in European populations, these properties are radically altered and the immediate impact presents itself as an increase in the potential efficiency of the Q-cycle and the ETC overall, resulting in increased longevity.

The cytbI7T polymorphism may be viewed in the context of caloric intake in order to better understand how a more efficient ETC could affect longevity in haplogroup H individuals. Caloric intake is essentially the ultimate input to the ETC, and the cytbI7T polymorphism may react differently with different levels of input. Haplogroup H individuals have significantly increased longevity during caloric restriction, but are not significantly different from haplogroup U individuals during other time periods, even during recent years of caloric over-consumption. The increased ETC efficiency of the haplogroup H individuals, which cytbI7T seems to be responsible for, is most advantageous during caloric restriction.

This could be for a variety of reasons. ROS leakage from the mitochondrial ETC is responsible for signaling apoptosis to the cell when the cell meets a certain threshold. An increase in Q-cycle and ETC efficiency, particularly under caloric restriction (sparse electron input), likely lowers the ROS leakage from the mitochondria and prevent more cells in certain tissues from undergoing apoptosis, thus increasing longevity in haplogroup H individuals. As we see no difference in longevity among haplotype H and U individuals during caloric over-consumption, this increased efficiency may not be of any advantage during these time periods because other factors may override the advantage. For example, a potential factor could be that of excessive electron input to the ETC as a result of hyper-calorie intake. Having a highly reduced ETC due to excessive electrons in the system may drastically increase the rate of ROS production, thus swamping the benefit haplogroup H individuals receive from cytbI7T. On the other hand, we suggest that during caloric restriction the benefit from cytbI7T plays a critical role on the precise efficiency of the ETC and increases longevity in haplogroup H as a result.

## Supporting Information

Figure S1 The phylogenetic tree of the 107 mammalian mitochondrial genome sequences (protein-coding portions) used as input for TreeSAAP.
(4.45 MB EPS)

Table S1 The GenBank accession numbers for the 107 mammalian cytb sequences used in this study.
(0.03 MB XLS)
